# What Counts as a Surrogate Decision?

**DOI:** 10.1177/10499091231168976

**Published:** 2023-03-30

**Authors:** Benjamin H Levi, Anne EF Dimmock, Lauren J Van Scoy, Theresa Smith, Bronson Kunzler, Andrew J Foy, Laurie Badzek, Michael J Green

**Affiliations:** 1 Department of Humanities, 12310 Pennsylvania State University College of Medicine, Hershey, PA, USA; 2 Nursing Sciences Building University Park, 15965 Penn State College of Nursing, Hershey, PA, USA

**Keywords:** advance care planning, surrogate decision, spokesperson, end of life, proxy, medical decision making

## Abstract

When patients lose decision-making capacity, others must make surrogate decisions on their behalf. What counts as a surrogate decision might seem self-evident. But as clinician-researchers in the field of advance care planning, we have found that it is not always so clear-cut. In this paper, we describe how and why this is a matter of concern, a novel approach for assessing whether a surrogate decision occurred, and findings from this assessment.

## Introduction

When patients lose decision-making capacity, others must make surrogate decisions on their behalf. In the context of advance care planning (ACP), a surrogate decision-maker is someone who is chosen (by the patient prior to losing decision-making capacity) or assigned (based on legal statute) to speak for the patient and represent their values, goals, and wishes when they cannot speak for themself. Implicit in any discussion or research involving surrogate decision-making is some common understanding of what constitutes a surrogate medical decision. At first blush, this might seem self-evident: a surrogate decision is a decision made by one person on behalf of another. But as clinician-researchers in the field of ACP we have found that, upon deeper reflection, many situations are far from clear-cut. In this paper, we explain the context in which this issue arises, along with its relevance; provide several examples that demonstrate the problem; describe a process developed by our research team to adjudicate what counts as a surrogate decision; and share our findings.

Many people in the United States lack decision-making capacity (DMC) at the end of life.^1^ DMC is a clinical determination defined by standard criteria,^2^ for which there is broad consensus.^3-5^ Adult patients are generally presumed to have DMC unless their behaviors or medical condition raise concerns—in which case medical or mental health professionals evaluate the patient’s cognitive/psychological abilities. Such presumptions are codified in both legal statutes and professional standards of care.^6-9^

When medical decisions need to be made for a patient who lacks DMC, family members are usually called upon to serve as spokespersons to make these surrogate decisions.^1,5^ Though ACP is often framed as an intervention to help ensure that *patients’* preferences are followed, a corollary aim of ACP is to help *spokespersons* be prepared to make surrogate decisions that align with what patients would have wanted.^1,10-17^ But to know whether surrogate decisions are aligned with patient wishes or whether surrogate decision-making is associated with any particular outcome, we must first agree on what counts as a surrogate decision. Clinicians and ACP researchers generally consider a surrogate medical decision to have occurred when someone makes a treatment-related decision on behalf of the patient—which may occur because the patient has requested this, is too weak to make the decision, or lacks DMC altogether.^8,18,19^ However, our research on ACP, as reported in Simmons et al.^20^ and Van Scoy et al,^21^ has shown that family members, or others positioned to serve as the patient’s spokesperson, sometimes have a different view of what constitutes a surrogate decision. In interviews we conducted with spokespersons for patients who experienced a serious medical event, multiple spokespersons did *not* consider the act of telling the medical team that their loved one would have wanted to be intubated or admitted to the intensive care unit to be a surrogate decision. In one case, the wife of a patient with severe heart failure reported that when she told the doctors to go ahead with a heart transplant, it was not a surrogate decision. In exploring this with her, she reported that the proposed treatment was really a “no-brainer” because there was “no real choice” in the matter, and therefore it did not constitute a decision at all. So, too, we found in Simmons et al.^20^ and Van Scoy et al.^21^ that spokespersons often drew little distinction between serving as an *advocate* for their loved one (for example, requesting that the doctor send the patient to a rehab facility rather than return home or calling 911 when their loved one was short of breath) vs acting as a *surrogate decision-maker*. Qualitative analysis provides evidence that family members of patients with severe chronic illness often have quite varied and overlapping perspectives on what advocacy means and how it is related to surrogate decision-making.^21^ To the extent these findings are generalizable, understanding this disconnect is important because, to accurately assess the impact of ACP on outcomes related to surrogate decision-making, we must first have an accurate and shared understanding of what constitutes a “surrogate decision”.^13,14,22-24^

It is also important to resolve this disconnect from a conceptual standpoint. In the absence of generally agreed upon definitions, operational problems arise in medical practice –whether one is deciding how many bacteria per high power field constitute a urinary tract infection, or if particular rules should apply to a family member’s treatment decisions.^8,25,26^ So, too, some fundamental reliance on consistency underpins any defensible system of reasoning and decision-making. Whether one embraces a deontologic, consequentialist, principalist, casuistic, or any other systematic approach to ethics, establishing shared understanding is key for conflict resolution.^27-29^ Of course, even the most hardy definitional agreement will not preclude interpretive variation –which is grounded in any number of cultural, regional, religious, age-related, or other differences in perspective. Yet the search for common ground fundamentally relies on the need for shared understanding.

There exists a large, well-established literature on the various permutations of *shared decision-making*,^30-32^ which occurs when a patient (or their spokesperson) along with their clinician deliberate and contribute to the decision-making process as a partnership. The norm for shared decision-making is that a capacitated patient retains ultimate decision-making authority and that when the patient lacks DMC those who make medical decisions on the patient’s behalf should do so in a manner that is consistent with the patient’s values/goals/preferences. That said, not everything done to benefit an incapacitated patient or promote their values/goals/preferences qualifies as a surrogate decision. Clear examples of this include providing money or other resources to help obtain the care a patient needs to survive, informing the hospital staff what kinds of food the patient likes, or running errands to help support the person’s family.

So, too, we know that there are a variety of considerations that influence the way decisions are made (or actions taken) to promote another person’s values/goals. Sometimes, we base our decisions/actions on what we think the patient would want. But sometimes, we decide/act based on what we think will best promote the other person’s values, even when we believe the other person would have chosen otherwise. The point is that we ought not simply equate *surrogate decision-making* with deciding/acting in a way that is consistent with that patient’s wishes. This, in conjunction with our research findings in Simmons et al.^20^ and Van Scoy et al.^21^ regarding the variability in how spokespersons interpret surrogate decision-making, led us to create a process for both exploring and adjudicating what should count as a surrogate decision, and why. In a forthcoming qualitative manuscript, we will detail how spokespersons interpreted surrogate decision-making and how this concept differed from the notion of advocacy. But here, our goal is to describe the process we created for resolving disputes as to whether a surrogate decision has been made and discuss the implications for clinicians and researchers.

## Materials and Methods

This work arose in the context of a broader research study on surrogate decision-making.^33^ The parent study was a randomized controlled trial comparing two ACP interventions with patients who had advanced cancer or severe heart, lung, or kidney disease.^33^ The primary outcome was whether the patient’s named healthcare spokesperson felt well-prepared for surrogate medical decisions that they made on behalf of their loved one (ie, self-efficacy to make surrogate decisions). After completing the ACP intervention, these spokespersons were called by a research coordinator every 12-16 weeks and asked to respond to a series of survey questions. Among these was the probe: “Have you had to make a major medical decision on behalf of your loved one?” If they indicated that they had made such a decision, a brief qualitative interview was carried out to learn more about the reported decision. In addition to field notes taken during the call, all conversations with spokespersons were recorded and transcribed. This study was approved by the institutional review board at Penn State University and registered at clinicaltrials.gov (NCT02429479).

The results of the most recent phone interviews were then discussed during a weekly research meeting attended by our study team, which included nurse- and physician-scientists, bioethicists, the research coordinator who conducted the interviews, a lawyer and sociologist, clinicians who treat severely ill patients, and several medical students involved in this project. When the team agreed that the reported event was, in fact, a surrogate decision (or if the patient had died, thereby precluding future surrogate decisions), the spokesperson was then scheduled for their third and final study visit—at which they would complete various study instruments and an in-depth, semi-structured interview. In carrying out this protocol, it quickly became apparent that what qualified as a surrogate decision was sometimes interpreted differently by spokespersons (compared to the research team) and that substantial disagreements also arose within our own team.

### Clinician-Researcher Consensus Process (CRCP)

**Figure 1.**
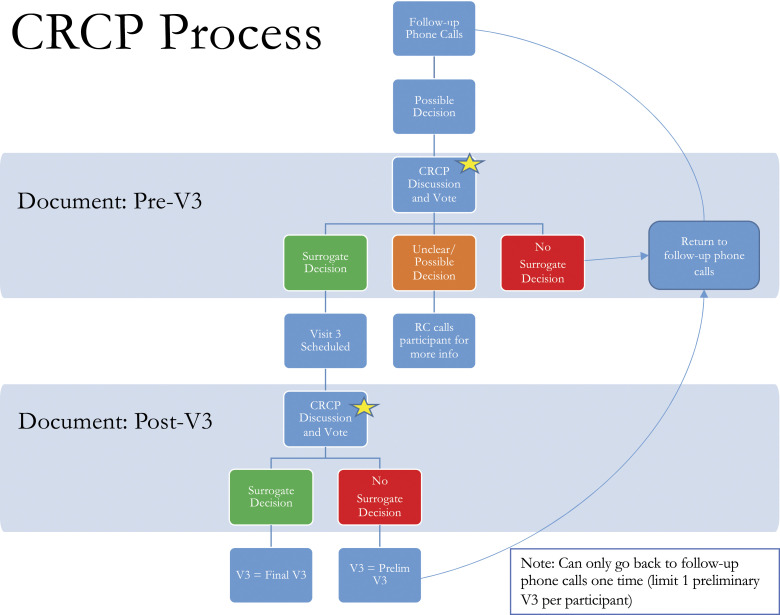
The clinician-researcher consensus process. (RC: Research coordinator ; V3: Visit 3)

In an attempt to better understand and systematically categorize disputed decisions, we devised the Clinician-Researcher Consensus Process (CRCP, see Figure 1). At weekly team meetings, the CRCP began with the research coordinator summarizing presumptive surrogate decisions (as identified by spokespersons and/or by the research coordinator) and the events surrounding them (as described by the spokespersons). Research team members discussed each case, and a preliminary vote was taken to gauge people’s initial interpretation of the events as: (1) a surrogate decision, (2) not a surrogate decision, or (3) unclear/need more information. Typically, the relevant section of the interview transcript was read aloud, sometimes the audio recording itself was played, and in the ensuing discussion, research team members would probe the available information to better understand what had happened. For example, on multiple occasions, a spokesperson reported that their loved one had been confused, was taken to the Emergency Department, and then released home without any decisions being made. But based on the study team’s understanding of conditions that might precipitate such Emergency Department visits (urinary tract infection, hypoxia, etc.), the CRCP would investigate what diagnostic testing and/or treatment had been carried out. Sometimes, this involved re-examining the interview or examining the patient’s electronic health record; other times, a brief follow-up interview with the spokesperson was recommended. In the case of a reported surrogate decision that involved a diagnostic test, the CRCP discussion explored whether the patient had decision-making capacity at the time of the “decision” (eg, whether to undergo a CT scan) and, if so, how the patient was involved. Did the medical team explicitly seek consent from the family member to obtain the scan or simply inform them that it was part of the standard in-hospital routine? These CRCP discussions were iterative, building off prior cases and shared areas of agreement, particularly with regard to what characterized “advocacy.”

Often, this CRCP process resulted in unanimous agreement that a surrogate decision had been made: a spokesperson reported having provided consent on behalf of their incapacitated spouse to be intubated and admitted to the intensive care unit, or a patient’s adult child reported having honored their parent’s wishes by declining life-sustaining medical treatment. But some case discussions were quite contested, prompting extensive deliberation. For example, while all CRCP members agreed that a spokesperson made a surrogate decision when they consented to have their incapacitated loved one placed in a long-term care facility, there was disagreement regarding whether it was a surrogate decision (vs. advocacy) when a hospitalized patient who was scheduled to be discharged to home instead wound up going to a long-term care facility because their loved one requested it. Likewise, there was dissension among CRCP members over whether a spokesperson made a surrogate decision when they overrode the wishes of their capacitated loved one to ensure that a given treatment was delivered.

At the close of discussion, CRCP members cast a final vote for whether the reported event(s) included a surrogate decision, and these results were then recorded in a REDCap database.^34^ When the CRCP reached a definitive conclusion, a third and final study visit was scheduled to conduct an in-depth interview with the spokesperson. In some cases, the CRCP determined that an additional prior phone interview was needed to gather more information. In other cases, a final study visit was scheduled, but with the caveat that if the in-depth interview suggested no surrogate decision had been made, the team would revisit the case to decide whether the spokesperson should return to the study pool for additional phone follow-up (until a surrogate decision was reported or their loved one died). When spokespersons were returned to the study pool, their in-person third study visit was classified as “preliminary” as opposed to the “final study visit” (see Figure 1).^35^ Of note, it was quite common for spokespersons to relay (and for in-depth interviews to confirm) that their loved ones had died without any surrogate medical decision having been made—a finding that runs counter to what is often reported in the published literature.^1,36,37^

## Results

Of the 114 spokespersons (Table 1) in this study whose telephone interviews underwent CRCP assessment, 5 were returned to the interview protocol for periodic phone calls, and 109 were brought in for a final visit (V3) to complete their participation in the study. For the 109 final interviews, there was consensus among all involved for 83.5% of cases (n = 91) that either a surrogate decision had been made (n = 50, 45.9%) or the patient had died without any surrogate decision being made (n = 41, 33.6%). But there were 18 instances (16.5%) where either the spokespersons reported a surrogate decision and the CRCP disagreed (n = 15, 12.3%; see cell C in Table 2), or the CRCP identified a surrogate decision and the spokesperson did not agree (n = 3, 2.8%; see cell B in Table 2). The CRCP discussions involved a mean of 6 research team members (range 4 to 8), and votes were unanimous in 79% (90/109) of CRCP decisions.

**Table 1.** Demographics of Spokespersons.

Characteristics	Family Caregivers (n = 114)
Gender, female n (%)	86 (75.4%)
Age,^a^ mean (SD), years	59.1^a^ (12.8)
Race/Ethnicity, n (%)	
Hispanic or Latino	3 (2.7%)
Black or African American	21 (18.4%)
White	87 (76.3%)
Did not respond	2
Patient disease category, n (%)	
Cardiac	31 (27.2%)
Pulmonary	25 (21.9%)
Cancer	39 (34.2%)
Renal	19 (16.7%)
Education, n (%)	
Some high school	1 (.8%)
High school graduate or GED	28 (24.6%)
Some college or technical school	45 (39.5%)
College graduate	24 (21.1%)
Graduate or professional school	16 (14.0%)
Current Marital Status, n (%)	
Never married	11 (9.8%)
Married	93 (81.8%)
Divorced or legally separated	7 (6.3%)
Domestic partnership	2 (2.0%)
Did not respond	1
Current Employment Status, n (%)	
Employed part-time	10 (8.8%)
Employed full-time	51 (45.1%)
Not currently employed and not seeking work	9 (8.0%)
Not currently employed but seeking work	3 (2.7%)
Retired	33 (29.2%)
Other	7 (6.2%)
Did not respond	1

^a^Age at the time of V3.

**Table 2.** Classification of Possible Decisions by Spokespersons and the Clinician-Researcher Consensus Process.

	Clinician-Researcher Consensus Process	
**Spokesperson**	Decision	No Decision
Decision	Cell A (n = 50)	Cell C (n = 15)
No Decision	Cell B (n = 3)	Cell D (n = 41)

Some of the narratives we encountered elicited disagreement within the study team about how surrogate decision-making should be categorized. One such case involved a hospitalized patient who was being discharged with a plan for at-home physical and occupational therapy. But just prior to discharge, the spokesperson of the incapacitated patient inquired whether the patient could instead go to a residential rehab facility, precipitating a change in the discharge plan. Several members of our study team considered the spokesperson’s intervention to be an act of advocacy rather than surrogate decision-making because the spokesperson was not responding to a treatment question but, instead, advocating for an option they perceived to be better. Others labeled this a surrogate decision because the patient’s care plan was changed as a result of the spokesperson’s actions. A similar disagreement arose among research team members with regard to a different situation, where a capacitated patient was taking a medication that was causing unpleasant side effects but did not want to “bother” their physician about it. The patient’s spokesperson felt differently and contacted the patient’s physician to report the side effects and to ask about changing the medication, which the physician did. Some team members felt this was a surrogate decision, while others did not. Another kind of disagreement that arose among study team members was whether merely conveying information (eg, what the patient explicitly said they would or would not want) constituted a surrogate decision. Some study team members argued that simply relaying what the patient said was no different than handing over a document, thereby making the spokesperson a messenger rather than a decision-maker. Others, however, argued that the paradigm of surrogate decision-making involves unadorned representation of the patient’s wishes. These team members argued that clinicians classically explain that the spokesperson’s job is to represent the patient’s wishes and values, and that this representation is more accurate when it is grounded in explicit conversations with patients themselves.

## Discussion

This article identifies several meaningful differences in how spokespersons, clinicians, and researchers understand the concept of surrogate medical decision-making. While prior ACP research has explored various factors related to surrogate decision-making (such as clinicians’ role in the process, the resources that spokespersons rely on, and whether surrogate decisions accurately represent patient wishes),^38-40^ to our knowledge this is the first study to identify that, compared to standard interpretations used by clinicians and researchers, some spokespersons have a very different understanding of what it means to make a surrogate decision.

At the very least, this has implications for how we assess interventions that focus on improving surrogate decision-making. Because valid conclusions rely on having shared definitions, it is important that researchers and clinicians take into account heterogeneity regarding what counts as a surrogate decision. However, our experience arriving at these findings also identified the need for conceptual clarification among clinicians and researchers themselves, not only to differentiate surrogate decision-making from advocacy but also regarding what should count as a surrogate decision.

As this issue pertains to good faith efforts by a spokesperson to support a patient’s wishes and goals, we could simply stipulate that to count as surrogate decision-making (as opposed to advocacy), a spokesperson must be acting on behalf of a patient who lacks decision-making capacity. So, in the example mentioned earlier, it would not count as a surrogate decision when a spokesperson arranged for a medication change for a capacitated patient who did not want to “bother” their physician about the medication’s unpleasant side effects. It is a bit more challenging, however, to find a non-arbitrary way of dealing with decision points that arise because a spokesperson is asking questions in an effort to advocate for a patient.

Consider again the case of the incapacitated patient being discharged from the hospital who could either go to a residential rehab facility or, instead, go home and receive home-based physical and occupational therapy. If the patient’s spokesperson is asked by a clinician to decide between these options, this clearly seems to be a surrogate decision. But should it also count as a surrogate decision if it was the spokesperson who raised the question to begin with? On its face, the answer seems to be yes, since the spokesperson is deciding (on the patient’s behalf) where they will be discharged. That said, the spokesperson is also acting as an advocate by pushing to identify the best treatment for the patient rather than simply accepting the option presented to them. Here, the spokesperson’s advocacy has *created the opportunity* to make a surrogate decision—which may help explain why spokespersons in our study co-mingled these terms.^20^ For this reason, it seems that the key issue is not how the treatment decision arose but whether there is a decision that the spokesperson is being asked to make.

As such, during our CRCP meetings, we considered question-asking, discussion of treatment options, and other forms of spokesperson engagement with the medical team to be precursors to actual decisions—and hence not part of surrogate decision-making, per se. But even with this sharper focus on actual decisions, we found substantive differences among research team members regarding how to interpret spokespersons’ involvement. This was particularly the case when attempting to distinguish “making a decision on a patient’s behalf” from simply “communicating the patient’s wishes.” In examining this distinction, we identified 6 variations for characterizing spokespersons’ involvement in the decision-making process:

(1) A spokesperson decides on behalf of a patient who lacks decision-making capacity (DMC).
(2) A spokesperson decides on behalf of a patient who has DMC but whose concerns or wishes are being ignored by their healthcare providers.
(3) A spokesperson decides on behalf of a patient who is believed to have DMC but cannot effectively communicate their wishes (to anyone).
(4) A spokesperson voices the decision that the patient has made but cannot clearly communicate to their healthcare providers.
(5) A spokesperson confirms the decision made by the patient because
  a. It’s not entirely clear whether the patient had DMC when they made the decision, or
  b. The patient’s healthcare providers want the spokesperson’s confirmation as further evidence that the patient’s decision should be followed.
(6) A spokesperson shares the patient’s (prior, capacitated) decision after the patient loses DMC.

For clinicians and researchers, the first variation is really the paradigm for surrogate decision-making and ideally involves using substituted judgment. The second variation *could* count as surrogate decision-making if the spokesperson is genuinely stepping into the role of decision-maker because the clinical team is disregarding the (capacitated) patient—which sometimes happens when there are major breakdowns in patient-clinician communication. What characterizes this variation is that the spokesperson becomes the decision-maker because the patient-clinician dynamic precludes continued patient participation. By contrast, the third variation occurs when a medical disorder (eg, advanced amyotrophic lateral sclerosis, locked-in syndrome) renders a capacitated patient unable to communicate their wishes to anyone, and the spokesperson must step in to make surrogate decisions. In the fourth variation, a capacitated patient again cannot effectively communicate with the clinical team, but because they can communicate with their spokesperson, the spokesperson is not actually making decisions. As such, the spokesperson’s role is that of an interpreter. In the fifth variation, the spokesperson is not so much making a decision as corroborating for the clinical team that the decision voiced by the patient is in keeping with what the spokesperson believes to be the patient’s wishes and values. Given the host of clinical factors that can affect a patient’s mental capacity or their ability to make an authentic decision, such reliance on spokespersons is both common and distinct from true surrogate decision-making.

The sixth and final variation only emerged after robust discussion revealed that even good-faith surrogate decision-making does not always involve substituted judgment. When surrogate decision-making does require substituted judgment, the spokesperson is expected to synthesize everything they know about the patient (their values, goals, wishes, fears, risk tolerance, resilience, etc.), apply that information to the situation at hand, and use their best judgment to reach a decision they (the spokesperson) think the patient would want. By contrast, we encountered multiple instances in which spokespersons were simply relaying a message, much as if they were handing over a document containing the patient’s expressed wishes. The distinction between these two variations turns on whether a spokesperson is having to interpret and project a patient’s values and wishes onto the current situation, or instead is merely parroting information. Clearly, if a patient has never expressed their wishes regarding how they would want to be treated in their current circumstance, substitute judgment will be needed. But even if they have expressed their wishes, the spokesperson will need to exercise some judgment if the patient’s wishes don’t readily map onto the current circumstances—which could be due to changes in prognosis or treatment options or changes in the patient’s value, goals, outlook, etc.

With these distinctions in mind, we think it could be helpful to categorize spokespersons’ actions in terms of whether they are simply relaying information vs exercising judgment. For spokespersons, doing this could help clarify their responsibilities as either messengers (variations 4, 5, and 6) or delegates who must use their knowledge, insight, and judgment (variations 1 and 3, and sometimes variation 2) to actually make a (surrogate) decision on behalf of the patient. Clinicians could use this binary categorization to help spokespersons better understand their role and to provide prompts or ask questions that help spokespersons carry out that role. For researchers, understanding these differences could be important for both developing and testing interventions designed to help patients and spokespersons prepare for momentous medical decisions. For several decades there has been an understanding that the role of a spokesperson is to represent the patient’s wishes. We hope that the current findings help clarify the various nuances of what that representation may or may not involve.

### Limitations

The spokespersons in our study may not be representative of surrogate decision-makers more broadly, particularly given that their loved ones had advanced severe illnesses, and they had all engaged in advance care planning. Although we endeavored to consider the range of clinical scenarios faced by a possible spokesperson, there may be circumstances that do not fit clearly into these categories. Additionally, the CRCP reflected the background, disciplinary biases, and decision-making preferences of the members of the study team; it is possible that a different group of CRCP members could have arrived at other conclusions regarding what constituted a surrogate decision. Future studies are needed to apply this paradigm to surrogate decisions in other research and clinical contexts.

## Conclusion

This investigation identified meaningful differences in how healthcare spokespersons, clinicians, and researchers interpret what it means to make a surrogate decision. We also identified six variations for how spokespersons represent their loved ones and propose that the variations that entail spokespersons having to exercise their judgment are true examples of surrogate decision-making. We suggest that this conceptual clarification is both important and useful for spokespersons, clinicians, and researchers.
